# Estimation of Residual Stress in Selective Laser Melting of a Zr-Based Amorphous Alloy

**DOI:** 10.3390/ma11081480

**Published:** 2018-08-20

**Authors:** Wei Xing, Di Ouyang, Ning Li, Lin Liu

**Affiliations:** School of Materials Science and Engineering, and State key Lab for Materials Processing and Die & mold Technology, Huazhong University of Science and Technology, Wuhan 430074, China; wxing@hust.edu.cn (W.X.); lliu2000@mail.hust.edu.cn (L.L.)

**Keywords:** selective laser melting, amorphous alloy, finite element analysis, residual stress

## Abstract

An accurate estimation of residual stresses is crucial to ensure dimensional accuracy and prevent premature fatigue failure of 3D printed components. Different from their crystalline counterparts, the effect of residual stress would be worse for amorphous alloys owing to their intrinsic brittleness with low fracture toughness. However, the generation of residual stress and its performance in 3D printed amorphous alloy components still remain unclear. Here, a finite element method combined with experiments and theoretical analyses was introduced to estimate the residual stress in selective laser melting of a Zr-based amorphous alloy. The results revealed that XY cross scanning strategy exhibits relatively low residual stress by comparison with X and Y strategies, and the residual stress becomes serious with increasing bar thickness. The residual stress, on the other hand, could be tuning by annealing or preheating the substrate. The above scenario is thoroughly understood according to the temperature gradient mechanism and its effect on microstructure evaluation.

## 1. Introduction

Amorphous alloys (also called metallic glasses, MGs) are a unique class of materials that possess an amorphous atomic-level structure and display a plethora of desirable mechanical, chemical, and physical properties, which makes them one of the most promising engineering materials [1,2,3,4,5,6]. However, the poor processability, combined with ambient-temperature brittleness, and limited size have been the Achilles’ heel to structural applications of BMGs [7,8]. Selective laser melting (SLM) as an additive manufacturing (AM) process, has been demonstrated as an effective technique to break through the bottleneck in processing amorphous alloys in recent years [9,10,11,12,13]. For instance, previous literature [14,15,16] reported that a full amorphous structure could be obtained by controlling parameters such as fast scanning rates, to avoid the risk of crystallization in the heat affected zone (HAZ). Therefore, SLM provides an opportunity to fabricate amorphous alloy components without dimensional or geometric limitations. Furthermore, the 3D printed amorphous alloy specimens also revealed excellent mechanical properties such as combined high strength and fracture toughness [13], exhibiting alluring prospects for near-future applications. However, steep temperature gradients caused by high heating and cooling rates during laser processing generally cause inhomogeneous thermal distribution, induce heterogeneous thermal expansions and contractions, and inevitably result in serious thermal and residual stresses [17]. The high stresses will trigger cracking, delamination, distortion and fatigue-failure in 3D printed components [18], especially for these amorphous alloys with intrinsic brittleness and low fracture toughness. Therefore, it is essential to understand the origin, distribution and evolution of residual stress in 3D printing amorphous alloys.

Residual stress has been widely investigated in laser processing crystalline metals, and various experimental methods [19,20,21] have been introduced to characterize residual stress in these 3D printed specimens. For example, mechanical methods including sectioning, contour, hole-drilling, ring-core, curvature, etc., were used to evaluate macro-stresses, while these methods only reflect residual stress in some local regions. Non-destructive “physical” methods, such as diffraction analysis, are more relevant for assessing residual stress at the grain level or at the atomic scale, but this “physical” method requires a very thin sample that is hard to print. Different from the above methods, the curvature method measures the deflection or curvature of a part caused by residual stresses, reflecting thermal stresses within layers. Therefore, this method is more suitable for 3D printed components, because SLM is based on the melting of successive layers, and the variation of processing parameters (such as scanning strategy, layer thickness, preheating, etc.) has a significant effect on residual stresses [22]. Accordingly, some researchers [23] used an overhang “cantilever” to investigate the influence of process parameters on distortion or to validate the distortion prediction model. Furthermore, the finite element method (FEM) was introduced to predict and reduce residual stress of SLM-fabricated components in recent years [24,25,26]. Alvarez et al. [27] established an FEM-model for divine distortion in a Ni-based alloy cantilever structure and to investigate the influence of meshing, layer activation and equivalent thermal loads on prediction capability and computational cost. Li et al. [28] developed a temperature-thread multi-scale modelling approach to predict residual stress and distortion of an AlSi_10_Mg twin cantilever manufactured by SLM. Parry et al. [29] built a thermo-mechanical model to analyze the effect of laser scanning strategy on the generation of residual stress in SLM-fabricated Ti-6Al-4V parts.

With respect to 3D printing amorphous alloys, the previous literature focused only on the possibility of 3D printing amorphous alloys [30,31], the effect of laser processing parameters on microstructure evolution [12,32,33], thermal stress induced micro-cracks [13,34,35] and possible mechanical properties [13,15,16]. The investigation of residual stress in 3D printed amorphous alloys is actually scarce, and the detailed distribution remains unclear, which is deleterious for controlling the dimensional accuracy and preventing premature fatigue failure of the 3D printed components. Therefore in this work, the finite element method combined with experiments and theoretical analysis was introduced to estimate the residual stress in selective laser melting of a Zr-based amorphous alloy. The results reveal that the XY cross scanning strategy exhibits relatively low residual stress by comparison with X and Y strategies, and the residual stress becomes serious with increasing bar thickness. The residual stress, on the other hand, can be tuning by annealing or preheating the substrate. These results provide a new route to improve the forming quality of 3D printed amorphous alloy components with a large scale and complex structure.

## 2. Constitutive Model and Finite Element Simulation

**Figure 1:**
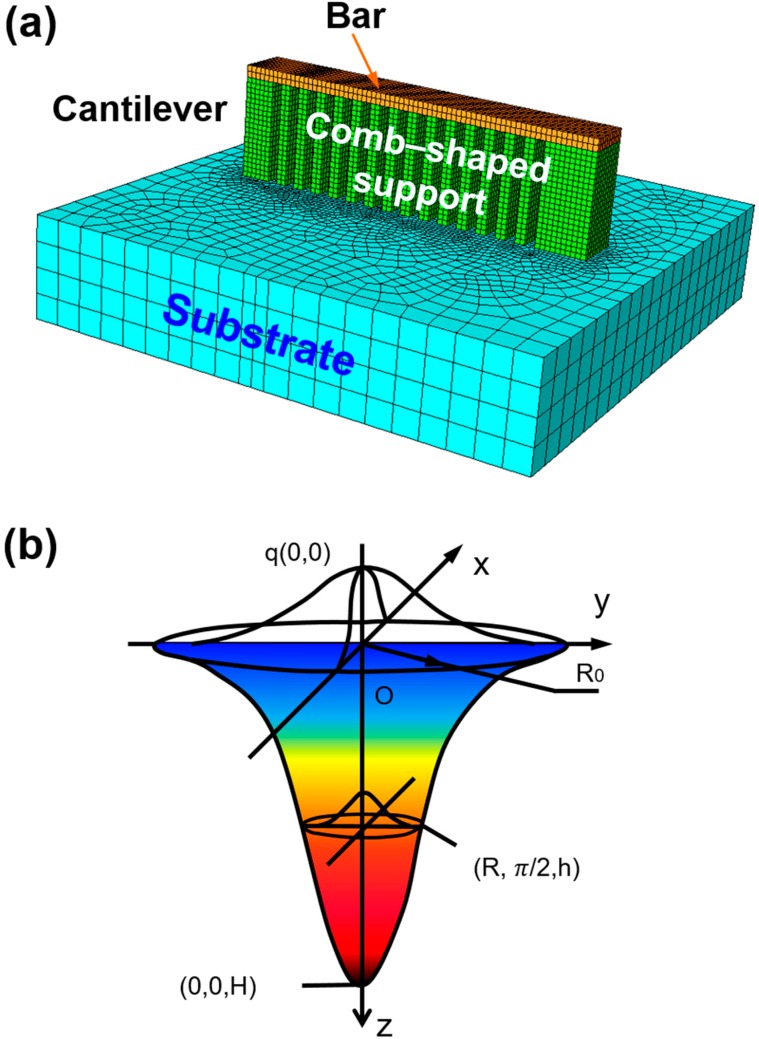
(**a**) Three-dimensional coupled thermo-mechanical finite element model and (**b**) representative heat input modeling with Gaussian distribution.

A thermal-mechanical coupled analysis model consisting of a cantilever and substrate was established by ABAQUS software (ABAQUS 6.10, Dassault, France), as shown in Figure 1a. Gaussian distributed moving heat flux was applied via an ABAQUS subroutine DFLUX. The moving direction and velocity of the heat flux were controlled by a subroutine, and elements were activated step by step sequentially as the heat flux moved (“birth and death” technique [36]). In order to reduce computational costs and predict the results well, Alvarez et al. [27] suggested a ratio between real manufacturing layers and model layer activation steps of 8 (48 real layers, activated in 6 steps). Because the distortions are mainly caused by the bars, only bars are activated in this model. The dimensions of the cantilever are designed as 3.75 mm (length of Y-axis) × 0.5 mm (width of X-axis) × 1.12 mm (height of Z-axis). Fine hexahedral elements with dimensions of 0.025 mm (width) × 0.025 mm (length) × 0.025 mm (thickness), similar to particle dimension, were used in experiment. In addition, the mesh size of substrate increased gradually from 0.025 mm to 0.25 mm along the X and Y-axes, but was maintained as 0.25 mm along the Z-axis. C3D8T elements were used from the ABAQUS element library for analysis. As a result, an acceptable finite-element model with a compromise in calculation accuracy and computation time is successfully established.

### 2.1. Heat Input Modeling

Considering the penetration depth in the powder bed during laser processing, a moving volumetric heat source assumed to obey a Gaussian distribution is designed to simulate the interaction between the laser and material, as illustrated in Figure 1b. The thermal flux density was applied to the powder layer using an ABAQUS subroutine DFLUX, expressed as [37]:

(1) $$q(x,y,z)=\frac{3c_{S}QA}{\pi H\left(1-\frac{1}{e^{3}}\right)}\exp\left[\frac{-3c_{S}}{\log\left(\frac{H}{z}\right)}\left(x^{2}+y^{2}\right)\right]\;$$

where *A* is the laser absorptivity of materials affected by the wavelength, *Q* is the laser power, *H* is the height of heat source, and $c_{S}=\frac{3}{R_{0}^{2}}$ is the concentric coefficient of heat-flux distribution of the cross section, in which $R_{0}$ is the effective radius of the heat source. The parameters used for finite element simulation are listed in Table 1. Furthermore, the latent heat of phase transformation should be considered, due to the process of melting and solidification of the material during SLM. The enthalpy (H) is defined as a function of temperature [38]:

(2) H=∫ρcdT 

### 2.2. Heat Transfer Modeling

In the SLM process, heat losses to the environment from the molten pool are determined through radiation, convection acting on the surface, and conduction into the support structure and substrate. Therefore, boundary conditions for heat transfer should be considered, described as [13]:

(3) $$q_{cond}=q\left(x,y,z\right)-q_{c}-q_{r}\;$$

where the heat losses through conduction $q_{cond}$, convection $q_{c}$ and radiation $q_{r}$ in the laser scan model are expressed as [39]:

(4) $$q_{cond}=-k\Delta T\;$$

(5) $$q_{c}=h_{c}\left(T-T_{0}\right)\;$$

(6) $$q_{r}=\sigma \in \left(T^{4}-T_{0}^{4}\right)\;$$

in which k is thermal conductivity, $h_{c}$ is the heat convection coefficient, σ is the Stefan-Boltzman constant, ∈ is the emissivity of the powder bed, 𝑇 is the temperature of the molten pool, and $T_{0}$ is the ambient temperature.

The transient spatial distribution of the temperature field during SLM can be depicted as follows [40]:

(7) $$\rho \frac{\partial \left(c_{p}T\right)}{\partial t}=\frac{\partial }{\partial x}\left(k\frac{\partial T}{\partial x}\right)+\frac{\partial }{\partial y}\left(k\frac{\partial T}{\partial y}\right)+\frac{\partial }{\partial z}\left(k\frac{\partial T}{\partial z}\right)+q\left(x,y,z\right)\;$$

in which ρ, k are the density and thermal conductivity, respectively, of the material, $c_{p}$ is the specific heat capacity, and T is the temperature. The temperatures of the powder bed and substrate are both set to ambient temperature as the initial condition and can be defined as [41]:

(8) $$T\left(x,y,z,t\right)|{}_{t_{0}=0,x,y,z\in D}=T_{0}=296K\;$$

### 2.3. Residual Stress Model

The moving laser inevitably generates a non-linear thermal gradient, and thermal-mechanical change promotes residual stresses and distortions. Therefore, based on the transient spatial distribution of the temperature field given by Equation (7), the temperature gradients that induced residual stresses can be described as:

(9) $$\sigma_{v}=\delta_{v}\lambda \varepsilon_{kk}+2\mu \varepsilon_{v}-\delta_{v}\left(3\lambda +2\mu \right)\alpha T\;$$

where λ and μ are the Lame constants related to elasticity modulus E and Poisson’s ratio v of the material and represent the deformation caused by the temperature gradient. The deformation dependent $\varepsilon_{v}$ can be expressed as:

(10) $$\varepsilon_{v}=\frac{1}{2}\left(\frac{\partial \mu_{i}}{\partial x_{j}}+\frac{\partial \mu_{j}}{\partial x_{i}}\right)\;$$

The elastoplastic model was adopted for residual stress calculation, and relevant data are acquired from the strains generated in the SLM process. The strains comprise elastic, plastic and thermal natures, defined as [29]:

(11) $$\varepsilon_{ij}=\varepsilon_{ij}^{e}+\varepsilon_{ij}^{p}+\varepsilon_{ij}^{th}\;$$

where $\varepsilon_{ij}$, $\varepsilon_{ij}^{e}$, $\varepsilon_{ij}^{p}$, $\varepsilon_{ij}^{th}$ are the respective total strain, elastic strain, plastic strain and thermal strain, determined by [42]:

(12) $$\varepsilon_{kl}^{e}=\sigma_{ij}^{e}\cdot E{\left(T\right)}^{-1}\;$$

(13) $$d\varepsilon_{kl}^{p}=d\lambda \frac{\partial f}{\partial \sigma_{ij}}\;$$

(14) $$\varepsilon_{kl}^{th}=\alpha_{ij}\left(T-T_{\infty }\right)\;$$

in which f is the flow area capability, $\alpha_{ij}$ is the coefficient of thermal expansion, $T_{\infty }$ is the reference temperature, λ is a constant that depends on the properties of the material; for a perfect elastic-plastic material, λ can be expressed as:

(15) $$\lambda =\frac{3GS_{ij}S_{kl}}{\sigma e^{2}}\;$$

### 2.4. Inherent Shrinkage Model

Here, we also consider the inherent shrinkage that is the main driving force for distortion during the cooling process [27]. The equivalent thermal strain should be accommodated by the part, which would lead to the redistribution of strains and stresses, described as:

(16) $$\varepsilon^{th}=\alpha \cdot \Delta T\;$$

where $\varepsilon^{th}$ is the equivalent thermal strain, α is the thermal expansion coefficient, ΔT is the temperature gradient. 

## 3. Experimental Procedures

**Figure 2:**
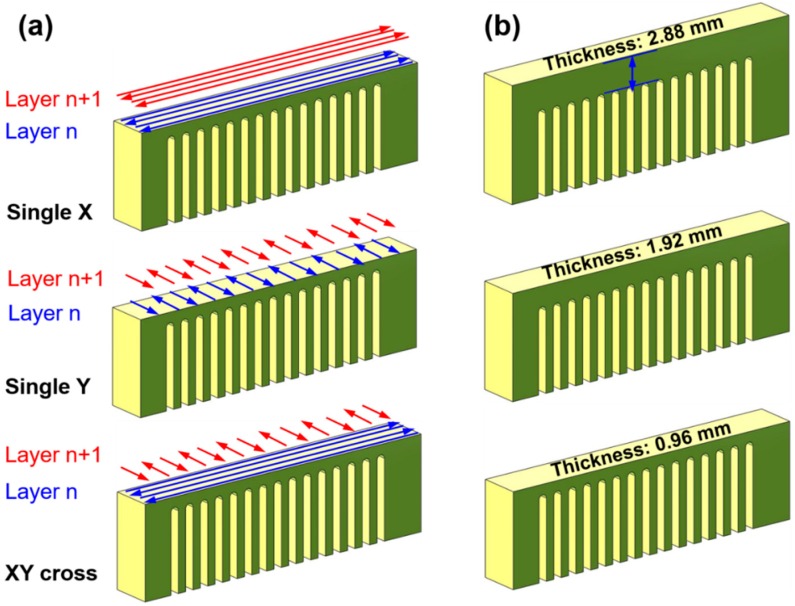
Schematic diagram of different (**a**) laser scanning strategies and (**b**) bar thicknesses.

The Zr_55_Cu_30_Ni_5_Al_10_ (at %) amorphous alloy powders used in this work were produced through high-pressure inert gas atomization. The physical properties of amorphous alloy are listed in Table 2. The SLM experiment was conducted using a commercial SLM machine (FORWEDO LM-120, Forwedo, Harbin, China) equipped with a Nd:YAG fiber laser. The SLM device generates a laser beam with focus diameter of 80 μm, maximum power of 500 W, and wavelength of 1060 nm. An inert high purity argon gas atmosphere with an oxygen content below 100 ppm after vacuum was used during the entire experiment. Here, optimized processing parameters with a laser power of 240 W, scanning speed of 1200 mm/s, powder layer thickness fixed at 60 μm and scanning space of 100 μm were adopted based on our previous experiments [35]. The cantilever structure was designed according to the FEM model. At the two ends of the cantilever arms, the additional supporting body was adopted to stand deformation. The bars were printed with different scanning strategies, such as X, Y and XY cross scanning strategies (scanning direction of 90° alternated among layers), as shown in Figure 2a. The other group of cantilever specimens was fabricated with the same XY cross scanning strategy, but for various bar thicknesses (Figure 2b).

## 4. Results

### 4.1. FEM-Simulated Residual Stress Field

**Figure 3:**
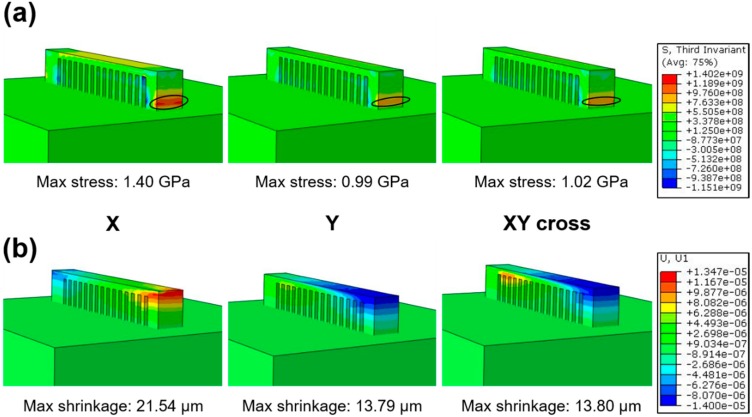
(**a**) Residual stress field and (**b**) longitudinal shrinkage under different laser scanning strategies.

To illustrate the residual stress distribution under different scanning strategies, the simulated contours of residual stress in cantilever parts are described in Figure 3. In general, the stress distribution (Figure 3a) and shrinkage (Figure 3b) in the cantilever components varied with scanning strategies, and the largest residual stresses are obtained in the connection region between the cantilever and substrate (as shown by the cycle in Figure 3a). It is worth noting that the measured shrinkage of the bar under the X scanning strategy is 21.54 μm, corresponding to the maximum residual stress concentration of 1.40 GPa. As for the Y scanning strategy, the shrinkage decreases to 13.79 μm with maximum residual stress concentration of 0.99 GPa. For the XY cross scanning strategy, the shrinkage of the cantilever is 13.80 μm, and the maximum residual stress concentration is 1.02 GPa, slightly different from the Y scanning strategy. Actually, the X and Y scanning strategies are identical; the significant difference in stress concentration and shrinkage between X and Y scanning strategies is caused by the cantilever structure. In order to build the cantilever part with lower residual stress accumulation, the XY cross scanning strategy is adopted in this work. 

**Figure 4:**
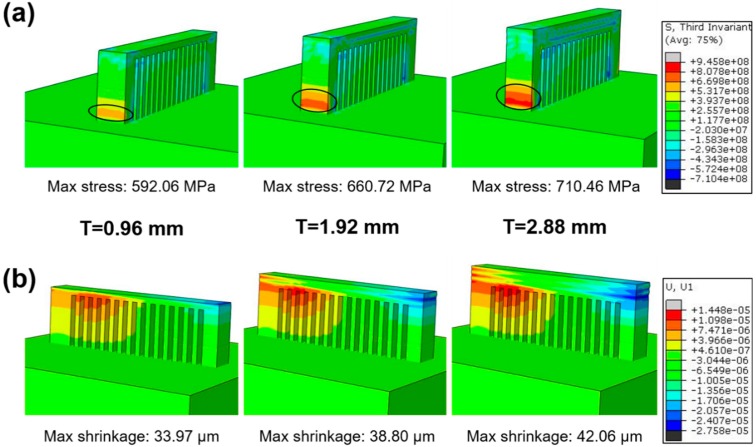
(**a**) Residual stress field and (**b**) longitudinal shrinkage of three typical bar thicknesses under the XY cross scanning strategy.

On the other hand, the shrinkage and the maximum stress concentration for bars with different thickness were also investigated, as described in Figure 4, from which the residual stresses are concentrated in similar locations (as shown by the cycle), and shrinkage varies with the thickness of the bars (Figure 4b). For the thin bar (thickness of 0.96 mm), the maximum shrinkage is 33.97 μm with a relatively low residual stress of 592.06 MPa. When the bar thickness increases to 2.88 mm, the maximum shrinkage rises to 42.06 μm, and the residual stress accumulates to 710.42 MPa. These results indicate residual stress is dependent on bar thickness.

### 4.2. Measured Residual Stress Distribution

**Figure 5:**
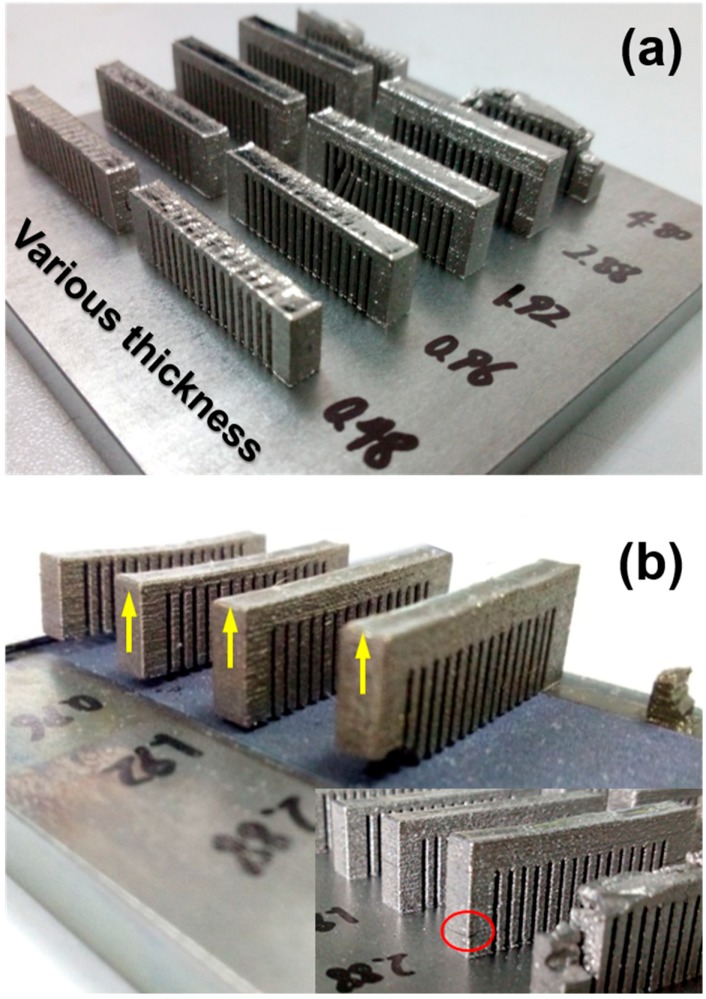
(**a**) 3D printed Zr-based cantilevers with various bar thicknesses and (**b**) spreading of cantilevers after separating the support.

On the basis of the previous research [35] and the above simulation, Zr-based amorphous alloy cantilever specimens with various bar thicknesses were 3D printed by SLM, as depicted in Figure 5a. The thickness ranging from 0.48 mm to 2.88 mm possibly indicates various residual stresses that will induce distinct distortion. To detect this difference, the comb-shaped supports were cut off from the substrate, and the cantilever bent towards the Z direction (building direction) due to the residual stress release (Figure 5b).

**Figure 6:**
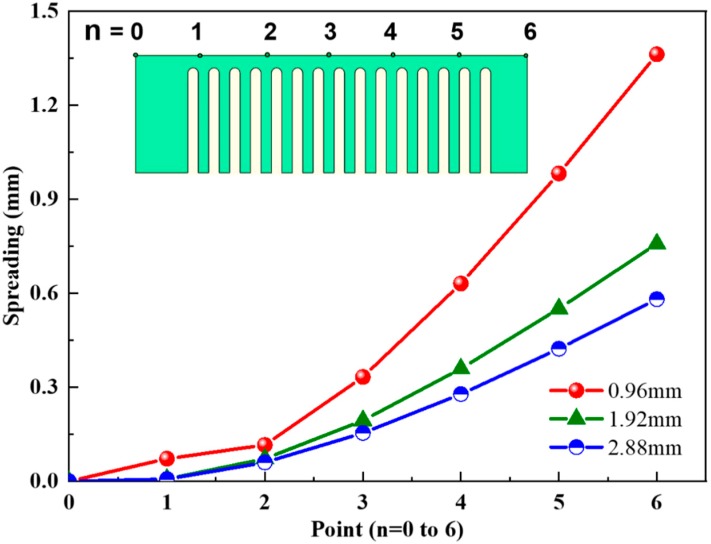
Spreading of the cantilevers depending on bar thickness and measurement point (n).

To further probe this disparity, the distortion of 7 typical points on the bar surface along the longitudinal direction was measured, and the results are summarized in Figure 6, wherein the distortion of cantilever became serious along the longitudinal direction (such as from point 0 to point 6). However, the degree of lifting varies with the thickness of bars. For instance, the maximum tilted height is 1.36 mm for a bar with thickness 0.96 mm; while when the bar thickness increases to 2.88 mm, the maximum deformation decreases to 0.58 mm.

**Figure 7:**
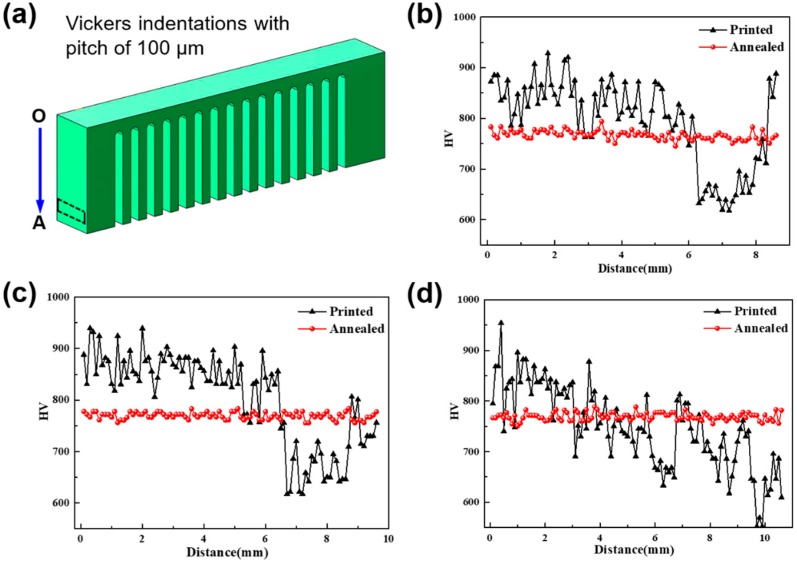
The Vickers hardness before and after annealing at 250 °C for 10 h along the OA direction (**a**) with pitch of 100 μm under the XY cross scanning strategy with bar thicknesses of (**b**) 0.96 mm; (**c**) 1.92 mm; (**d**) 2.88 mm.

Owing to the fact that the heat affected zone (HAZ) contains a mixed structure of the amorphous phase and some nanocrystals with dimensions of 100–200 nm as mentioned above, the nanoindentation test [43] is not suitable to measure the residual stress. In order to demonstrate different residual stresses induced by various processing parameters, Vickers micro-indentation was introduced here to measure the hardness along the lateral face with a mirror finish, as shown in Figure 7a. Vickers indentations had an interval of 100 μm, peak load of 2.94 N and dwell time of 10 s. Figure 7b–d illustrates the measured hardness of SLM-fabricated samples with various bar thicknesses. When the bar thickness is 0.96 mm (Figure 7b) and 1.92 mm (Figure 7c), the approximate hardness is about 850 HV, but it decreases to about 600 HV in the region (as shown by the rectangle in Figure 7a) of residual stress concentration (decrease of about 29.4%), identical to the FEM simulated results (Figure 4a). On the other hand, the hardness continuously decreased to 550 HV similar to the SLM-fabricated sample with a bar thickness of 2.88 mm, possibly owing to the residual stress that increased continuously along the OA direction in the relatively large affected zone. 

### 4.3. Residual Stress Released after Annealing and Preheating

**Figure 8:**
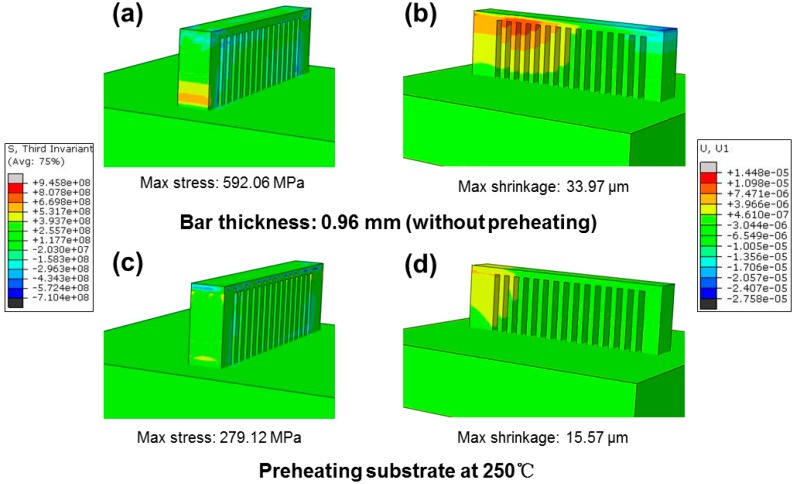
(**a**) Residual stress field and (**b**) longitudinal shrinkage of the bar with a thickness of 0.96 mm under the XY cross scanning strategy compared with preheating at 250 °C (**c**) corresponding residual stress field and (**d**) corresponding longitudinal shrinkage of the bar.

To probe the possible methods to release residual stress as mentioned above, low-temperature annealing as a relatively economical method was first considered here. For comparison, micro-hardness was also tested after vacuum-annealing at 250 °C for 10 h; in this case the amorphous structure remains [44]. The measured hardness of all samples with different bar thicknesses are almost 770 HV, as shown in Figure 7b–d, demonstrating that annealing is an effective method to alleviate the residual stress. Furthermore, preheating of the substrate was also introduced here, shown in Figure 8. The boundary condition of a constant temperature field of 250 °C was installed for the manufactured cantilever with a bar thickness of 0.96 mm. It is worth noting that by preheating, the maximum residual stress concentration decreases from 592.06 MPa to 279.12 MPa, accompanied by shrinkage of bars from 33.97 μm to 15.57 μm.

## 5. Discussion 

**Figure 9:**
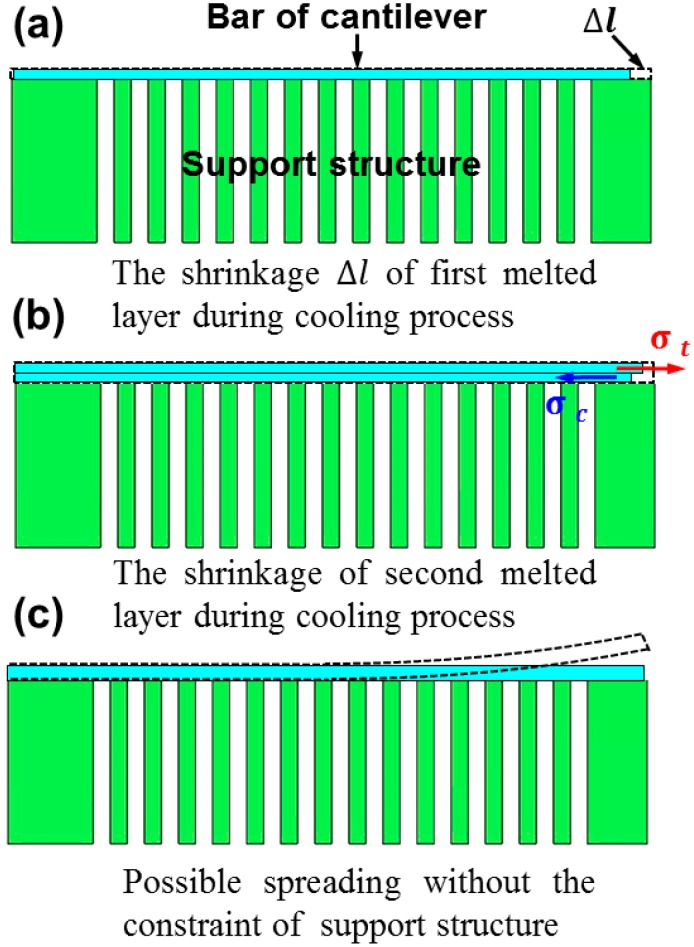
Schematic description of shrinkage of the (**a**) first and (**b**) second melted layer during the cooling process of the bar in the cantilever structure; (**c**) constraint of the support structure during deformation.

During the SLM process, steep thermal gradients generate around the molten pool and result in inhomogeneous shrinkage during fast cooling, which inevitably triggers high residual stress. To thoroughly understand the effect of processing parameters on residual stress, the shrinkage of the printed material should be determined, as illustrated in Figure 9. Owing to the temperature rise in flash laser heating, the bar material (assumed as the first layer) expands and then shrinks in all sections in the subsequent cooling process. When a cantilever is built with increasing bar thickness, the second layer cannot freely shrink due to the constraint of the previously solidified layer. Therefore, the shrinkage of the upper layer is relative small. In this case, the lower layer is compressed while the upper layer is lengthened [23], which generates a large tensile residual stress in the upper layer, causing distortion of the fabricated components, as illustrated in Figure 9. On the other hand, the constraint of the comb–shaped support structure causes a stress concentration nearby the connection region between the cantilever and substrate (as shown in Figure 4a). Therefore, the residual stress increases with bar thickness, based on the FEM simulation and shown in Figure 4a, which should induce relative serious distortion of the sample. While from Figure 6, the distortion of the cantilever became moderate with increasing bar thickness, attributed to the considerable section modulus [23]. 

**Figure 10:**
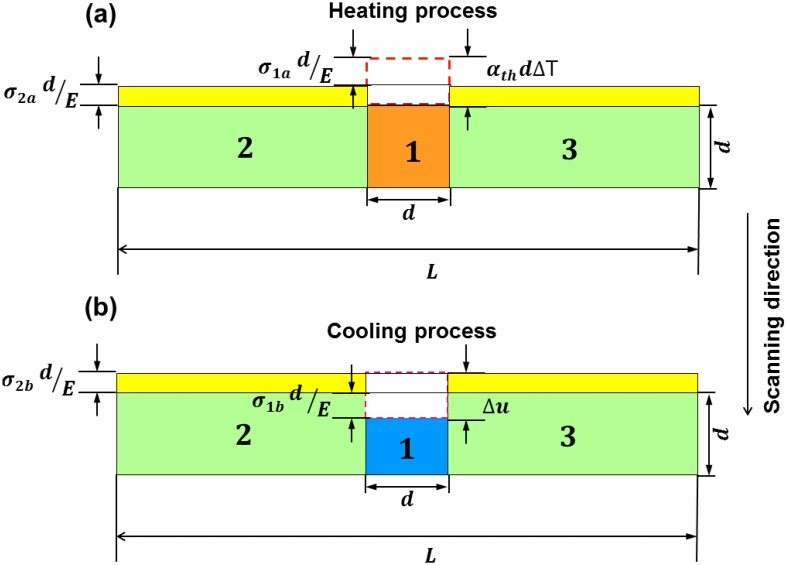
Schematic diagram of heating deformation of the previous layer during laser scanning of the current layer.

The above phenomenon can also be understood in detail, as shown in Figure 10a, the heated zone is assumed with width and depth of *d* (zone 1), wherein zones 2 and 3 are normal-temperature zones. During the laser processing, the instantaneous temperature of zone 1 is *T*, while the temperature in zones 2 and 3 remains $T_{0}$ (such as ambient temperature). In this case, the free thermal expansion of zone 1 can be defined as $\alpha_{th}d\Delta T$ ($\alpha_{th}$ is the coefficient of thermal expansion), which indicates that there is compressive stress in zone 1, but tensile stress in zones 2 and 3. Considering the constraint of zones 2 and 3, the shortening value σ1adE of zone 1 should be considered; therefore, the total expansion value of zone 1, Δμ=αthdΔT+σ1adE ($\Delta T=T-T_{0}$ is the temperature gradient between zone 1 and zones 2 and 3; $\sigma_{1a}$ is the compressive stress of zone 1), and the total expansion value of zones 2 & 3 is σ2adE ($\sigma_{2a}$ is the tensile stress of zones 2 and 3); therefore,

(17) αthdΔT+σ1adE=σ2adE 

In addition, the compressive force of zone 1 is expressed as [45]:

(18) $$\sigma_{1a}S_{1}=-\sigma_{2a}\left(S_{2}+S_{3}\right)\;$$

where $S_{1}$, $S_{2}$ and $S_{3}$ are the cross-sectional areas of zones 1, 2 and 3, respectively, and $S_{2}=S_{3}$. According to Equations (17) and (18), $\sigma_{1a}=-\frac{2\alpha_{th}E\Delta TS_{2}}{S_{1}+2S_{2}}$, $\sigma_{2a}=\frac{\alpha_{th}E\Delta TS_{1}}{S_{1}+2S_{2}}$, and Δμ=σ2adE=αthdΔTS1S1+2S2.

After cooling, zone 1 is under tensile strain, while zones 2 and 3 are compressed, as described in Figure 10b. The tensile strain of zone 1 is σ1bdE ($\sigma_{1b}$ is the tensile stress of zone 1), and the final compressive strain of zone 1 is Δv=Δμ−σ1bdE. The final tensile strain of zones 2 and 3 is σ2bdE ($\sigma_{2b}$ is the compressive stress of zones 2 and 3), accordingly,

(19) Δμ−σ1bdE=−σ2bdE 

The tensile force of zone 1 should be equal to the compressive force of zones 2 and 3:

(20) $$\sigma_{1b}S_{1}=-\sigma_{2b}\left(S_{2}+S_{3}\right)\;$$

According to Equations (19) and (20), $\sigma_{1b}=\frac{2\Delta \mu ES_{2}}{d\left(S_{1}+2S_{2}\right)}$, $\sigma_{2b}=-\frac{\Delta \mu ES_{1}}{d\left(S_{1}+2S_{2}\right)}$, and $\Delta v=\frac{\alpha_{th}d\Delta TS_{1}^{2}}{{\left(S_{1}+2S_{2}\right)}^{2}}$. Therefore, the dependence of the compressive value of the heated zone on the temperature gradient with a closed region can be correlated through Δv∝ΔT.

When the sample is SLM fabricated with a single X scanning strategy, the heat flux direction is similar because of the parallel laser tracks. Therefore, a large temperature gradient along the building direction (Z direction) was generated in the molten pool by the substrate heat sink and the Marangoni flow [46]. Since the scanning velocity reaches 1.2 m/s, the other temperature gradient along the X direction should also be considered. These steep temperature gradients trigger high residual stress parallel to the X and Z directions. Similar residual stress fields overlapped layer by layer, finally accumulate severe residual stress. On the contrary, when the sample is fabricated by the XY cross scanning strategy, the heat flux direction rotates with the rotation of the laser tracks among successive layers. The rotation of laser tracks changes the residual stress field layer by layer. In this case, partial residual stress is counteracted, resulting in the reduction of the integrated residual stress (Figure 3). According to the Temperature Gradient Mechanism (TGM) [47,48], substrate preheating can reduce the temperature gradient in the molten pool and heat-affected zone, which alleviates the shrinkage of the bar and the residual stress.

The above results also present a micro-hardness dependent residual stress as shown in Figure 7b,c. The FEM results reveal that these local regions suffered high residual stress concentration exhibit tensile stress on the surface, which can be well understood according to the stress-induced dilatation or softening in amorphous alloys [49].

In general, when the applied stress is sufficiently high, dilatation or softening happens owing to the fast free volume creation rate than the annihilation rate [50,51]. Flores et al. [52] investigated the effect of the stress state on strain localization and proposed a relationship between the stress and the average free volume, namely, residual stresses induced volume dilation as free volume change. Under a tensile mean stress, all dilatations are attributed to the change in free volume, and the hard sphere atomic volume is held constant: $v_{f}^{T}=v_{0}+\Omega \frac{\sigma }{B}$, where, $v_{0}$ is the average initial volume assigned to each atom without a superimposed stress, Ω is the atomic volume and B is the bulk modulus. In this work, the maximum tensile stress is 592 MPa (bar thickness of 0.96 mm); therefore, the reduction of hardness ($\Delta H_{T}$) can be illustrated by [53]:

(21) $$\frac{\Delta H_{T}}{H_{0}}=\frac{v_{f}^{T}-v_{0}}{v_{0}}=\frac{\sigma \Omega }{Bv_{0}}\approx 31.2\%\;$$

It is interesting that the calculated result (31.2%) is comparable to the experimentally measured micro-hardness reduction (29.4%), demonstrating the validity of the above analysis. Accordingly, annealing treatment would reduce the concentration of defects through free volume annihilation, which causes relative dense packing and results in the increase of hardness. Furthermore, the tensile stress and compressive stress balance each other, inducing homogeneous distribution of hardness as shown in Figure 7. 

## 6. Conclusions

In summary, the residual stress during selective laser melting of a Zr-based amorphous alloy was investigated, based on both experimental and theoretical results, and the following conclusions can be drawn. 

(1) Experiments combined with finite element simulation revealed that the XY cross scanning strategy exhibits relatively low residual stress by comparison with the X and Y strategies, and the residual stress becomes serious with increasing bar thickness. The residual stress, on the other hand, could be tuned by annealing or preheating the substrate.

(2) The theoretical analysis suggested that reducing the thermal gradient by the XY cross scanning strategy and preheating the substrate could enhance homogeneous shrinkage during fast cooling, which moderates the residual stress.

## Figures and Tables

**Table 1:** Parameters used for finite element simulation.

Parameter	Value
Laser power, P (W)	240
Scanning speed, V (mm/s)	1200
Density of Zr-based MG powder, ρ (kg m^−3^)	4129
Latent heat of fusion (J Kg^−1^)	2.638 × 10^5^
Melting point of Zr-based BMG (K)	1165
Coefficient for the heat convection, h (W m^−2^ K^−1^)	20
The Stefan-Boltzmann constant, δ (W m^−2^ K^−4^)	5.67 × 10^−8^
Radiation emissivity, ε	0.77

**Table 2:** Thermal physics parameters of the Zr-based amorphous alloy.

Temperature T (°C)	Specific Heat Capacity c (J Kg^−1^ °C^−1^)	Thermal Conductivity k (W m^−1^ °C^−1^)
20	326	4.9
100	346	6.2
200	342	7.4
300	271	6.8
400	233	6.6
500	−323	7.6
600	112	7.7
700	362	10.1
800	375	9.6
